# Vaccination: a look at the social representations of Brazilian children

**DOI:** 10.3389/fpubh.2024.1434513

**Published:** 2024-12-06

**Authors:** Suelen de Gaspi, Carlos Alberto de Oliveira Magalhães Júnior, Rosa Branca Tracana, Eduarda Maria Schneider, Graça S. Carvalho

**Affiliations:** ^1^Federal Institute of Paraná, Goioerê, Brazil; ^2^State University of Maringá, Maringá, Brazil; ^3^CI&DEI-ESECD-IPG, Guarda, Portugal; ^4^Federal Technological University of Paraná, Campo Mourão, Brazil; ^5^CIEC, University of Minho, Braga, Portugal

**Keywords:** central core, immunization, science teaching, anti-vaccination, health

## Abstract

**Introduction:**

The pandemic caused by COVID-19 has accentuated the debate on the need for vaccination and called into question the need to increasingly bring this topic, which is widely disseminated in the scientific world, to school classes at all schooling phases. In this scenario, science education plays a key role in disseminating knowledge about the importance of vaccination and the impacting factors of a lack of immunization. In order to better understand this movement, it is necessary to understand the representations of individuals as a way of broadening paths to change this scenario.

**Objectives:**

This study aimed to identify and analyse Brazilian primary school children’s social representations of vaccination.

**Methods:**

Using the free word recall technique, the term “vaccination” was applied to evoke children’s ideas. The analysis of co-occurrences of evocations permitted us to identify their representations’ centrality.

**Results:**

The results showed that the centralizing elements guiding these children’s social representations were “needle,” “pain,” and “health center.”

**Conclusion:**

These results show the need to prevent the phobia of needles that arises in the first vaccination experiences and reinforce the importance of discussing the subject of vaccination in science teaching. This issue is even more critical, given the spread and impact of “fake news” on social media. There is an increasing need to emphasize the importance of vaccination, not only as a factor of individual protection but also as a commitment to collective health. This study also showed that the school, as usual, must become an ally in tackling this reality with children and their families.

## 1 Introduction

### 1.1 The origin of vaccination and its social and educational implications

It was a long time before humans discovered a cure for certain diseases through vaccination. The first human vaccine did not emerge until the 18th century through the doctor Edward Jemmer during the smallpox outbreak (1). His discovery of immunization was criticized and mistrusted by many medical scholars and scientists. It was only overcome when his studies showed the smallpox vaccine’s effectiveness. Since then, society has realized that strengthening people’s immune systems is an important part of any health programme. Jemmer, in 1789, gave the health sector a relevant step forward in developing other vaccines (2, 3).

However, despite the proven benefits to human and collective health, many European movements emerged in the 1900s against vaccination (4). One justification was that the idea of compulsory mass vaccination would violate the principle of individual liberty, which was one of the ideological pillars of the 18th-century French Revolution. Similarly, in Great Britain, in 1860, the Anti-Vaccination League and Compulsory Vaccination raised doubts about the efficiency of the smallpox vaccine itself (4). The fight against disease relies not solely on society’s complex understanding of science but on each person’s understanding (5). This condition has led many social organizations, particularly those that manipulate social masses, to promote mistrust about the reliability of scientific experiments, discoveries, and results. Its effects vary according to society’s structure, considering its political, social, and cultural contexts.

The controversial issue of vaccinating people also reached the US Supreme Court in 1900. In 1905, the US government authorized many states to create a set of actions to vaccinate their population and have the vaccination instrument incorporated into public health policy (4).

Just as the vaccine was criticized worldwide, it was no different in Brazil. From the imperial period (starting in 1982) onwards, the country began to have developmentalist ideals in favor of industrialisation and the expansion of cities. The Brazilian political play to building a modern nation saw several social movements that are important for understanding the historical context of the Brazilian vaccination system, such as the Vaccine Revolt in 1904 (6). Since then, a lot has happened, and nowadays, the vaccination issue has become part of the routine of world society and Brazilian society, primarily due to the COVID-19 pandemic. Since the vaccine revolt and intense health struggles, from the 1970s onwards, Brazil has seen relevant public policies in defense of individual and collective health, such as the birth of the “National Immunization Plan” (7).

Despite the availability of essential vaccines to protect children and adolescents, Brazilian society has been slow to recognize the importance and effectiveness of vaccination. For this reason, the federal government, together with the states and municipalities, created policies that reinforced the compulsory nature of vaccination as a factor in accessing various social benefits.

Among the policies instituted to expand access to the vaccination schedule was the birth of the “Child and Adolescent Statute,” which made it compulsory for children to be vaccinated in the cases recommended by the health authorities. This instrument would make vaccination a right for children and young people (8). Although many controversies can lead to very conflicting scenarios about vaccination in Brazil, vaccination should not be seen as an obligation but as a declared right for the “Child and Adolescent Statute” itself; however, there is no question about this. Parents who do not follow the vaccination schedule established by the Ministry of Health can be held responsible.

Perceived as an important public health process, the vaccination process should be current not only in the health field but also in the education field. The pedagogical understanding of the subject must articulate a set of educational actions and guidelines to strengthen the theme, especially in Science Education. In this sense, one of the objectives highlighted in the Brazilian “National Curriculum Parameters” for the 4th and 5th grades of Fundamental Education is to “Identify the body’s natural and stimulated defenses (vaccines)” (9). In the “National Curriculum Parameters” referring to the contents for the subject of Natural Sciences, in the thematic block “human being and health,” it is emphasized that:

[…] it is possible to treat the immune system as the body's natural defense mechanism, which can be stimulated by vaccines, against the action of foreign elements. The variety of vaccines, their correct usage, modes of operation, and the importance of vaccination campaigns can be investigated through interviews with health agents at healthcare centers in the region.

[…] establishing connections between body health and the existence of natural and stimulated defenses (vaccines) (9).

This concern in Science Education is also reinforced by the compulsory Brazilian National Common Curricular Base, considering that by the end of Elementary Education, students should be capable of understanding the role of the State and public policies, especially on relevant aspects in health education such as vaccination campaigns, family and community health care programs, investment in research, awareness campaigns about diseases and vectors (10).

Indeed, building knowledge becomes important to counter distorted concepts about science, as has happened by some public authorities and other parts of society regarding vaccination (11). With school-based campaigns, students armed with knowledge and skills can build solid and reliable references to strengthen the field of science and become disseminators of scientific knowledge beyond the classroom walls (11).

## 2 COVID-19, “fake news” and social representations

Despite the historical context reinforcing the importance of the immunization process and the debate on the subject in the most diverse social contexts, the play of interests of dominant groups has meant that the production of knowledge has often responded to issues of power and capital accumulation and has put scientific production in check, as well as spreading misinformation, such as the case of the Vaccine Revolt in 1904, which raised doubts about the effectiveness of the vaccine, not unlike what happened in a recent context with the COVID-19 vaccine by the Brazilian Federal Government (6, 12).

The advent of social media has amplified movements that cast doubt on the efficacy of vaccination, promoting the spread of so-called ‘fake news’ through these platforms. This has led to the daily dissemination of news and information riddled with misinformation, particularly about health and vaccination. This trend, lacking a scientific basis, has significantly harmed public health, especially by influencing individuals not to vaccinate (13).

Saraiva and Faria (11), in 2019, state:

[…] Fake News has affected the most diverse areas of people's lives, from politics to public health. Recently, fake news about the Poliomyelitis and Triple Viral vaccines and their supposed relationship with autism fueled campaigns called the Anti-Vaccine Movement, in which parents of newborn children claimed that they refused to vaccinate their children. The scale of what happened was so grand that it triggered the reappearance of diseases already eradicated, with cases in Europe, the United States and Brazil.

Such movements cast doubt on the vaccine’s efficacy and cause a drop in the number of immunized individuals in the country. Indeed, seven of the nine vaccines indicated for babies had the worst coverage rates in Brazil in 2019, at least since 2013 (14). The Ministry of Health in 2020 also reported that none of the vaccination coverage targets available under the “Childhood Immunization Programme” were met (15). In this respect, the Butantan Institute (16) recognizes that eradicated diseases can return due to a lack of immunization.

All the above encouraged us to better understand this process by understanding people’s representations of vaccination. For this purpose, the Theory of Social Representations (SR) was applied. Social representations are part of ways of life, relationships, and individual or collective issues. Through social change, SR interpret concepts emerging from a historical context (17).

This Theory of SR was proposed by Moscovici in 1961 in his book “Psychoanalysis: Its Image and Its Public” (18). He understood that scientific and technological knowledge from thinking, dominant and elitist groups should not be prioritized, i.e., popular knowledge should also be the subject of SR study. In this sense, how one would try to explain the world and social objects would not only bring as a response a reproduction and duplication of concepts by society but rather how it reconstructs and represents the scientific knowledge in its daily life (19). Moscovici (20) explains that:

[…] social representation is an organized corpus of knowledge and one of the psychic activities from which men make physical and social reality intelligible, insert themselves into a group or into a daily exchange relationship, and liberate the power of their imagination.

This Theory has four main approaches (21): (i) Cultural/Anthropological and Sociogenetic, with Moscovici (20) as precursors; (ii) Structural Approach proposed by Abric; (22) (iii) Societal/Sociodynamic by Doise (23); and finally, (iv) the Dialogical, as proposed by Marková (24). In the present study, we embraced the structuralist approach (ii), which seeks to understand how social representation structures (central nucleus and peripheral system) are organized and how individuals use them to make sense of the world around them. This structuralist approach, originating in the 1970s, “was developed from the hypothesis that suggests that every representation […] is organized in such a way that, at its Centre, are the elements that give meaning to this social representation” (25). From this perspective, every social representation is organized around a Central Nucleus (CN) and a peripheral system (PS). The CN is represented by the group’s collective memory (26).

Therefore, the CN carries the group members’ shared values, beliefs, behaviors, and actions towards a certain object and is the fundamental element in the structuralist-based SR study approach: “[…] it is the change of this core that will indicate a change in the SR, just as it is the difference between cores that allows us to characterize two social groups as distinct in relation to an object” (21).

Considering that SR allow us to understand how people think, act, and feel and how they symbolize their social context in the face of a certain object, we understand it as an important way to understand the vaccination issue. Given the seriousness of the topic regarding individual and collective health and the widespread need to discuss vaccination in the educational context, this study aims to investigate the children’s social representations in the early grades (5th grade) of Fundamental Education on the vaccination topic. This group’s choice is anchored in Moscovici (27), who assumes that young children already have direct contact with SR, as they are immersed in social phenomena like adults. Furthermore, Barra Nova (28) elucidates that when belonging to the same social group, children can carry relevant information about the context in which they are inserted and about the elements that make up their subjectivity. As members of the collective, we understand the importance of children as “active agents in society, who build their own representations and, at the same time, contribute to the production of the adult world” (28), which, therefore, makes them very suitable to the present study.

## 3 Methodology

A sample of 20 students from a 5th grade classroom of a municipal school in the north-western region of Paraná, Brazil, was selected to study children’s social representations of vaccination as they can communicate through writing and are social subjects who “interact with people and institutions, react to adults and develop strategies to participate in the social world” (28). When asked for authorization to conduct this research, the Municipality’s Education Department suggested this school. Subsequently, the parents’ authorization was also requested, and the participants agreed to participate voluntarily. For the collection of SR, the free word evocation technique (29) was used to evoke free terms about “Vaccination” (30). This technique was not just chosen but meticulously selected because the most representative elements of an SR are the most accessible to consciousness and, therefore, easier to access (21, 31, 32). Thus, students were asked individually, without any reference material or dialogue with their peers and teacher, to write down the first five words that came to their mind regarding the term “Vaccination” and then rank them from one to five, with number one being the most relevant and number five being the least relevant. This process was not just a step but a crucial contribution to the respondents reassessing their choice of promptly evoked terms. Finally, the students were instructed to justify the choice of each term in a short text to “give analytical density to the evocations” (33).

This data collection technique is based on verbal expression to understand what the subjects verbalize, i.e., what is verbalized is important, as well as the order in which it occurs (21). Furthermore, the term order provides the level of salience as the more promptly a word is evoked, the greater its representativeness will be within its social group (34).

After the data collection, the evoked terms were organized into spreadsheets and divided into semantic groups. Words evoked only once and that did not fit into any group were discarded as they were not considered relevant for the representativeness of the group (35). The data were then subjected to analysis using the Iramuteq software. Initially, the prototypical analysis was conducted to identify the Central Nucleus of the social representation based on Abric’s structural approach (22). The software performs a cross between Frequency (F) and the Mean Order of Evocation (MOE), the result of which composes the four-box frame (36). This Abric’s methodological approach enables the comparison of the central nucleus of different social groups on the same object (21).

Indeed, prototypical analysis is the first phase of a deeper analysis of word co-occurrence and organization that allows for identifying the CN of a social representation (37). However, despite its relevance, it is recommended to use complementary analyses that may present more refined and conclusive results (21, 34, 37, 38). Among the possibilities for confirming the centrality of the elements, the similarity analysis (39, 40) was carried out, also with the Iramuteq software assistance. This technique was developed by Flament and pointed out by Sá (29) as “the main technique for detecting the degree of connectedness of the various elements of a representation.”

Thus, similarity analysis helps understand the connections between the evoked terms arranged in the CN based on their associative capacity; the maximum similarity tree displays vertices and edges that interconnect them (41). The circular images represent the vertices whose radius illustrates the frequency of each evoked term. The larger the frequency of the evoked term, the larger the vertex (radius). The connection between the terms is demonstrated through the edges, indicating the co-occurrence value, that is, the number of times the respondents mentioned two terms together (the thicker the edges, the higher the correlation between the terms).

The association of prototypical and similarity analyses enables the understanding of the terms that present greater symbolic value for the researched social group and, in turn, expresses the term polysemic character, which can have different meanings in different SR due to the psychological, political, historical, cultural, or social nature to which the message content is associated (41).

## 4 Results and discussion

The sample of 20 students from the 5th grade of primary school comprised twelve boys and eight girls aged between 9 and 12.

From the children’s evocations of the inductive term “Vaccination,” 86 words were recorded, of which most of the children (13) evoked the requested five words, but some did less: one child wrote 4 words, five children 3 words, and one child only 2 words. The words evoked were analysed semantically and then submitted to Iramuteq software. The collection of words evoked for the term “Vaccination” generated 16 groups, whose mean Frequency (F) was 4.44 and the Mean Order of Evocation (MOE) was 2.75. The Vergès Diagram was constructed using these values, delimitating the four quadrants with their respective groups that comprise the SR (Table 1).

**Table 1:** Elements of the social representations of 5th grade students regarding the inductive term “Vaccination.”

Central elements - 1st quadrant			Intermediate elements - 2nd quadrant		
High F and low mean order of evocations			High F and high mean order of evocations		
*F* ≥ 4.44 MOE < 2.75			F ≥ 4.44 e MOE ≥ 2.75		
Semantic group	F	MOE	Semantic group	F	MOE
Needle	9	2.6	Healthcare professional	6	3.2
Pain	9	2.3	Syringe	5	5.3
Protection	8	1.9			
Health Centre	6	2.7			

Intermediate elements - 3rd quadrant			Peripheral elements - 4th quadrant		
Low F and low mean order of evocations			Low F and high mean order of evocations *F* < 4.44 e MOE ≥ 2.75		
F < 4.44 e MOE < 2.75					
Semantic group	F	MOE	Semantic group	F	MOE
Importance	4	2.50	Cure	4	2.8
Health	3	1.70	Medicine	3	3.3
Vaccine	2	1.00	Fear	3	3.3
			Cotton	3	5
			Dizziness	2	4
			Agony	2	4
			Sickness	2	3.5

Table 1 shows the groups of words that make up the CN because they are the most readily evoked and accessible to the subjects (34). When grouped semantically, the “Needle” group represents the words evoked by children who readily associate the vaccine with inserting a needle into the body. This group was among the most evoked words, with a frequency of 9 and an OME of 2.60. The subsequent group, “Pain,” had an F of 9 and an OME of 2.30.

These two most readily evoked groups associate vaccination with the act of “getting needles” and the feeling of discomfort caused by this action. This relationship is evidenced through these extract examples: “The needle is to put the vaccine inside our body”; “The needle is to pierce my arm”; “The needle injects the liquid into my body.”

Similarly, in children’s conceptions, the term vaccination is also associated with “Pain.” This interpretation can be better understood in these quotes: “It hurts to get vaccinated”; “When I get vaccinated, my arm hurts a lot”; “Some vaccinations hurt a lot.”

This greater frequency of the elements “needle” and “pain” led us to consider that the children associate the act of being vaccinated with a traumatic moment motivated by their anxiety. Indeed, children often respond in advance with anxiety and panic prior to the vaccine, which can increase suffering (42).

Furthermore, part of this behavior can be stimulated by their parents. In fact, a report by Target Saúde (43), based on an American study, indicates that there is a strong relationship between children’s anticipated fear of vaccination and their parents’ behavior, which sometimes influences their fear long before vaccination. If not worked out in childhood, this fear can also contribute to non-vaccination in adulthood (43).

This scenario was the basis for our proposal to understand children’s representations of vaccination by learning about their shared ideas, thoughts, images and knowledge of a group, in other words, whether children are influenced by their socio-cultural context. If the role of social representations is also to situate objects, people and events in the context of a community (44), then by knowing children’s understanding of their consensual universe, it is also possible to understand part of the context shared by adults and the school environment in which they live.

Also included in the possible CN are the “Protection” group, with a frequency of 8 evocations and an OME of 1.90, and the “Health Centre” group, with a frequency of 6 and an OME of 2.70. Despite having a lower frequency than the previous groups, these two groups indicate that children understand vaccination as the “Protection” of life, as suggested by the following statements: “The vaccine protects us from the flu”; “The vaccine is important to protect us from dengue fever”; “You have to take it to be safe.” For the “Health Centre” group, the justifications refer to the geographical area or localization where they undergo the vaccination process.

This nuclear composition may strongly relate to the historical, social moment that society was sharing with the Covid-19 pandemic. However, although the children recognize that the vaccine is important, part of their justifications associate the act of immunization with some common diseases, such as the flu and dengue, as shown in the example above, with little regard for the fact that the vaccine also protects against various diseases throughout life, from childhood to old age.

This situation is also clear in the passage “Vaccines are to protect children,” as if protection is only aimed at a specific age group and not society as a whole. This misconception can be gradually changed through scientific literacy that promotes an understanding of immunization as a social importance. “The topic of vaccines can be very stimulating for science teaching and opens up space for discussion of various topics from a multidisciplinary perspective and inserted into the lives of students” (45).

**Figure 1:**
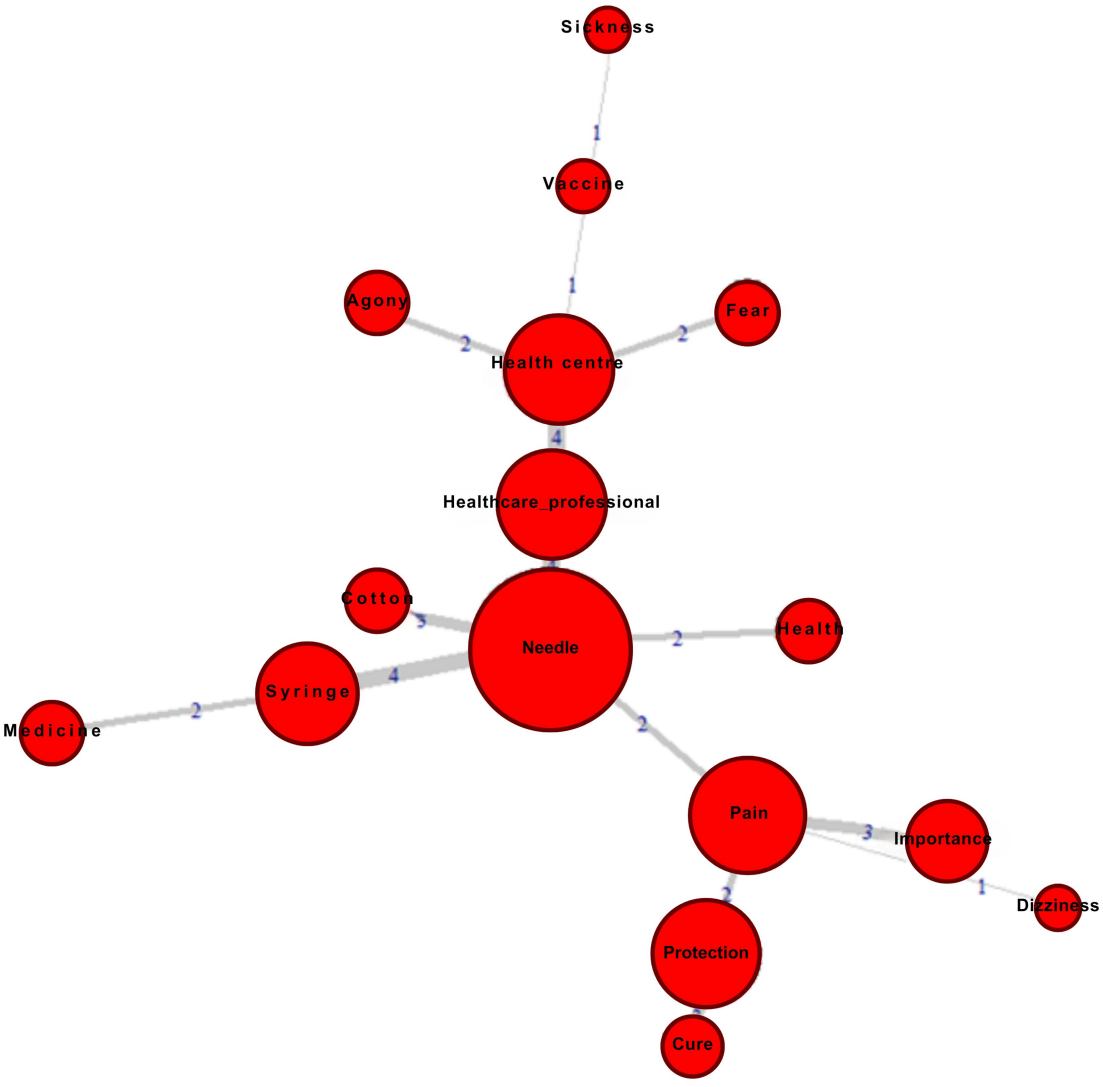
Maximum similarity tree of students in the 5th grade of primary school for the inducing term “Vaccination”.

To check the centrality of the elements in Table 1, data were subject to Similarity Analysis, where the maximum tree is displayed in Figure 1. This analysis aimed to help deepen the study of the representational structure (46). Furthermore, this strategy also acts as a “real proof” of the four-house table in an illustrative way (21).

As expected, the similarity analysis also showed that the centralizing elements of children’s social thinking were “Needle,” “Pain,” and “Health center,” with the element “Needle” standing out as a link that relates to the other blocks. In contrast, the “Protection” element, although having a high frequency compared to the other three elements and being in the CN of the prototypical analysis, does not manifest centrality because it does not give rise to new branches (39).

Based on both analyses (prototypical analysis and similarity analysis), it becomes clear that the children’s SR “Protection” group focuses particularly on the “act” of being vaccinated and the environment surrounding this moment (needle, pain, and health center) and not on the importance of vaccination itself for individual and collective health.

This situation raises important questions such as: are we effectively educating our children about the importance of vaccination? After all, if the act of getting vaccinated is more widely represented than the importance of getting vaccinated, then it is up to the school, through scientific knowledge via science teaching and health education, to address and change this process right from the early primary school years.

Indeed, regardless of age, vaccinations are part of everyone’s life, and everyone lives (or should live) with the vaccination experience. In this context, Cunha and colleagues (45) state:

[…]what else do people know about vaccines? Do they know how vaccines work in our bodies? And do they know how the scientific knowledge that culminated in the invention of the vaccine was constructed? How can the topic of vaccines be helpful for science education? […] school is a privileged place to start building this path.

Therefore, everyone, regardless of age, must have the scientific knowledge to maximize their understanding of the importance of vaccination to the detriment of the process. Promoting health education is crucial for placing children in a social context that guides them to implement health protection measures and can turn them into information agents in their family environment (47).

## 5 Final considerations

Many challenges have been faced throughout history in the scientific, social, political and technological fields so that today’s society can have access to vaccines. However, despite the historical context, there are still families who prefer not to vaccinate their children. This sad reality has led to the reappearance of previously eradicated diseases and an increase in the number of unimmunized people around the world. This situation has been maximized following the advent of social media and the spread of the so-called “fake news” on vaccination.

Therefore, it is necessary to understand this process better and what guides it. This is why this study aimed to investigate the social representations of a social group of children about “Vaccination.” The results allowed us to understand that children carry representations suggesting the act of being vaccinated rather than the importance of vaccination for health. These results reinforce the importance of discussing the topic of vaccination in the school context, especially in science teaching, especially in the face of the explosion of social networks and the spread of false news.

In addition, schools need to become allies in tackling this reality with children and their families and reinforce the importance of vaccination, not only as an individual protection factor but also as a commitment to collective health and social transformation.

## Data Availability

The original contributions presented in the study are included in the article/supplementary material, further inquiries can be directed to the corresponding author.
